# A Systematic Review of New Imaging Technologies for Robotic Prostatectomy: From Molecular Imaging to Augmented Reality

**DOI:** 10.3390/jcm12165425

**Published:** 2023-08-21

**Authors:** Severin Rodler, Marc Anwar Kidess, Thilo Westhofen, Karl-Friedrich Kowalewski, Ines Rivero Belenchon, Mark Taratkin, Stefano Puliatti, Juan Gómez Rivas, Alessandro Veccia, Pietro Piazza, Enrico Checcucci, Christian Georg Stief, Giovanni Enrico Cacciamani

**Affiliations:** 1 Department of Urology, University Hospital of Munich, 81377 Munich, Germanythilo.westhofen@med.uni-muenchen.de (T.W.); christian.stief@med.uni-muenchen.de (C.G.S.); 2 Department of Urology, Klinikum Mannheim, 68167 Mannheim, Germany; karl.kowalewski@googlemail.com; 3 Urology and Nephrology Department, Virgen del Rocío University Hospital, Manuel Siurot s/n, 41013 Seville, Spain; ines.rivero.belenchon@gmail.com; 4 Institute for Urology and Reproductive Health, Sechenov University, 117418 Moscow, Russia; marktaratkin@gmail.com; 5 Department of Urology, University of Modena and Reggio Emilia, 42122 Modena, Italy; stefanopuliatti@gmail.com; 6 Department of Urology, Hospital Clinico San Carlos, 28040 Madrid, Spain; juangomezr@gmail.com; 7 Urology Unit, Azienda Ospedaliera Universitaria Integrata Verona, 37126 Verona, Italy; a.veccia88@gmail.com; 8 Division of Urology, IRCCS Azienda Ospedaliero-Universitaria di Bologna, 40138 Bologna, Italy; pietropiazza1209@gmail.com; 9 Department of Surgery, Candiolo Cancer Institute, FPO-IRCCS, Candiolo, 10060 Turin, Italy; checcu.e@hotmail.it; 10 USC Institute of Urology, University of Southern California, Los Angeles, CA 90007, USA; giovanni.cacciamani@med.usc.edu

**Keywords:** robotic prostatectomy, imaging, new technologies, augmented reality, molecular imaging

## Abstract

New imaging technologies play a pivotal role in the current management of patients with prostate cancer. Robotic assisted radical prostatectomy (RARP) is a standard of care for localized disease and through the already imaging-based console subject of research towards combinations of imaging technologies and RARP as well as their impact on surgical outcomes. Therefore, we aimed to provide a comprehensive analysis of the currently available literature for new imaging technologies for RARP. On 24 January 2023, we performed a systematic review of the current literature on Pubmed, Scopus and Web of Science according to the PRISMA guidelines and Oxford levels of evidence. A total of 46 studies were identified of which 19 studies focus on imaging of the primary tumor, 12 studies on the intraoperative tumor detection of lymph nodes and 15 studies on the training of surgeons. While the feasibility of combined approaches using new imaging technologies including MRI, PSMA-PET CT or intraoperatively applied radioactive and fluorescent dyes has been demonstrated, the prospective confirmation of improvements in surgical outcomes is currently ongoing.

## 1. Introduction

Imaging technologies play a pivotal role in surgical interventions. The major applications include preoperative staging to assess local tumor invasion, metastatic spread and planning for intraoperative proceeding including 3D models for practice. Intraoperatively, orientation on anatomical landmarks provided by conventional imaging as well as virtual reality application for real-time detection of tumors and metastatic spread might be applied [1,2].

Robotic assisted radical prostatectomy (RARP) is the standard of care for localized prostate cancer [1]. Due to anatomical conditions of the pelvis, important nerve and vessel structures and difficulties detecting lymph node metastases, RARP is a challenging procedure [3]. Imaging technologies are therefore warranted to support the surgeon and to improve outcomes of patients. Interestingly, robotic procedures are prone to visual enhancement as an endoscopic camera is already used. Augmented reality and simulations are therefore an obvious to implement addition [4].

During prostatectomy, preoperative planning is paramount either for the primary tumor and its surrounding structures as well as for lymphatic spread into locoregional lymph nodes (LN). Here, 3D reconstructions and prostate models might help to visualize extraprostatic tumor growth and neurovascular invasion. As 3D models might help understanding of anatomical positions, they might support surgical planning. Furthermore, those reconstructions might be then used to overlay with the video console intraoperatively to guide the surgeon. Further, in conventional surgery, beta and gamma probes are used to detect metastases marked by radioligands, for example. Those technical solutions require adoption for robotic surgery. Interestingly, RARP is already using imaging through the video console. Therefore, combination of this approach with modern imaging technologies might be one of the cornerstones of modern RARP.

The present study is a systematic review of new imaging technologies for RARP that focuses on the preoperative planning and intraoperative utilization of technology as well as teaching modalities specific for RARP.

## 2. Materials and Methods

A systematic literature analysis was conducted on 24 January 2023. Pubmed, Web of Science and Scopus database were systematically queried with a predefined research string defined as followed:

((robotic prostatectomy) AND (augmented reality)) OR ((robotic prostatectomy) AND (molecular imaging)) OR ((robotic prostatectomy) AND (neuronal imaging)) OR ((robotic prostatectomy) AND (virtual reality)) OR ((robotic prostatectomy) AND (new imaging technology). All identified papers were considered for further analysis.

First, all duplicates originating from the three databases were removed. All identified studies from the three databases were then analyzed for overall eligibility. Therefore, only original articles were included and replies, editorials, reviews and book chapters were removed. The received articles were then screened for inclusion criteria. Inclusion criteria were then original articles covering any aspect of new technologies specific for RARP that either support imaging of the primary tumor or lymph node metastases to improve surgical outcomes or that focus on imaging and visualization for training modalities. Accordingly, we excluded articles that reported preoperative imaging without direct intraoperative utilization (screening via ultrasound or MRI, staging imaging including PSMA-PET CT imaging), preclinical models or technology for salvage lymphadenectomy. The focus was put on research published within the last 5 years. However, the literature research was conducted without restriction of the publishing year. The analysis for eligibility was performed independently by two researchers. In cases of disagreement, a third researcher was involved to form consensus. The protocol of this systematic review was not registered prior initiation of the study.

Analyses were performed according to the PRISMA guideline for systematic reviews [5].

## 3. Results

**Figure 1:**
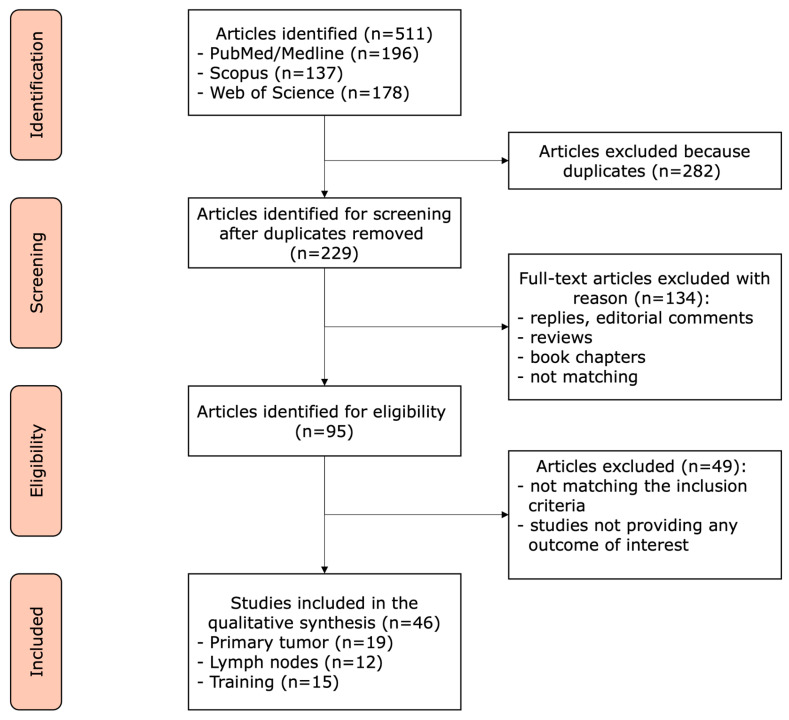
PRISMA flowchart.

The systematic search on Pubmed, Scopus and Web of Science with the described research string revealed 511 studies. After removing duplicates, 229 studies were screened. Here, 95 studies were identified for eligibility. Next, 49 studies were excluded for not meeting the inclusion criteria. A total of 46 articles were selected after qualitative analysis for this review (see Figure 1).

All 46 studies were analyzed and a level of evidence was determined based on the 2011 Oxford Center for evidence-based medicine level of evidence [6]. All studies were then categorized by the assessed organ (prostate, LN, abdominal wall), the area of application (preoperative planning, visualization of the primary tumor, intraoperative diagnostics, intraoperative detection of LN, education/training and feedback) as well as by the applied imaging modality (see Table 1).

Below we report our findings stratified by the imaging modalities for the primary tumor and locoreginonal LN detection as well as applications for training purposes.

### 3.1. Primary Tumor

From the identified 46 studies, 19 studies focused on imaging of the primary tumor either for preoperative planning, intraoperative tumor detection, real time imaging or to support intraoperative diagnostics.

#### 3.1.1. Preoperative Planning

Four studies focused on preoperative planning. Shirk et al. conducted a randomized trial (*n* = 92) to evaluate the performance of surgeons when reviewing virtual models prior RARP. The trial revealed improved oncological outcomes with significantly lower postoperative detectable PSA (31% vs. 9%, *p* = 0.036) and a trend towards lower positive margin rates. Surgeons changed their surgical strategy in 32% of the cases based on the reviewed model leading to a trend towards bilateral nerve sparing [8]. In addition, a retrospective analysis performed by Checcuci et al. revealed the use of a 3D model as a protective factor for positive surgical margins [10]. Similar results have been shown by Martini et al., that compared patients before and after introduction of 3D models derived from 3T-MRI [9].

When looking at the patient perspective, Wake et al. revealed that patients gain a better understanding of their disease when their organ was 3D printed versus visualized in augmented reality or viewed on a 3D or 2D screen [7].

#### 3.1.2. Intraoperative Tumor Detection and Real Time Imaging

A total of 12 studies focused on intraoperative real-time monitoring or augmented reality regarding the primary tumor.

Samei et al. demonstrate the feasibility of real-time augmented reality-based motion tracking of the prostate using ultrasound [13]. The working group has developed their system further and have tested the combination of preoperative MRI and ultrasound guidance in twelve patients undergoing RARP. Thereby, the surgeon can navigate the transducer via the robotic instruments. Imaging data are then overlayed on the endoscopic image. An accuracy of 3.2 mm is achieved [19].

A phase I study by Kratiras et al. using a tablet-based image guidance system that mapped the preoperative MRI to the patient revealed that such solutions are mainly used during challenging steps of RARP at the bladder neck and apical dissection as well as during nerve sparing [14].

Mehralivand et al. present a virtual reality imaging technology derived from preoperative MRI imaging that can be overlayed at several time points of RARP. However, according to the authors this system shows the limitation of VR-imaging, that it is not integrated into the video console as it was not considered useful for challenging surgical situations [17].

Schiavina et al. used MRI derived 3D models of the prostate that are superimposed on the video stream of the robotic system. The research group aimed to evaluate the impact of this technological support on intraoperative nerve sparing planning during RARP. The initial surgical plan was changed in 38.5% of all patients with 11.5% of all patients presenting with positive surgical margins after surgery. The sensitivity of the model was 70%, the specificity was 100% and the accuracy 92% [20].

Similarly, Porpiglia et al. demonstrate the feasibility of augmented reality RARP with a model accuracy of 1–5 mm with 85% of mismatch being less than 3 mm [11]. This group further tested hyperaccuracy 3D reconstruction with similar results [16]. In another study, Porpiglia et al. used an elastic augmented reality model to detect areas of capsular involvement during the nerve-sparing phase of RARP. This model has been developed to superimpose images even during the dynamic phases of surgery with deformation of the prostate. The authors demonstrate a superiority compared to 2D cognitive RARP in terms of detection of capsular involvement [15].

Although most research groups use MRI as input data for the augmented or virtual reality models, Canda et al. also incorporate PSMA-PET-imaging data for their VR model. The model was used in five RARP cases and revealed the clinical feasibility of this approach [18].

Intraoperative real-time augmented reality assistance might be achieved by deep-learning approaches and requires computing power. Therefore, Tanzi et al. investigate different algorithms to achieve this and demonstrated the superiority of a new convolutional neural network they applied with an intersection over unit (IoU) of 0.894 [21]. Similarly, Padovan et al. achieve real time 3D model alignment by semantic segmentation and use convolutional neuronal networks and motion analysis to compensate for rotation. Here IoU scores greater than 0.80 were achieved [22].

When evaluating the surgeons′ view on this development of augmented reality RARP, surgeons revealed a strongly positive opinion about this support for all evaluated critical steps of a RARP including bladder neck dissection, nerve sparing and apex dissection [12].

**Figure 2:**
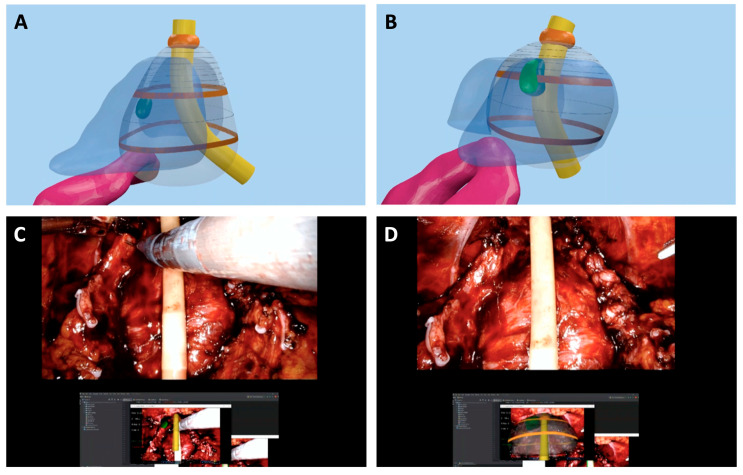
Augmented reality application for RARP. (**A**) 3D model of the prostate with intraprostatic lesion (**C**) 3D model of the prostate with lesion with capsular contact (bright green). (**B**,**D**) AI 3D-AR RARP: the 3D virtual model of the prostate was automatically overlapped to in vivo anatomy thanks to the AI; then a 3D guided selective biopsy was performed (images are courtesy of Prof. Porpiglia).

An example of the potential application of virtual reality superimposing video console real-time imaging is provided in Figure 2.

#### 3.1.3. Intraoperative Diagnostics

Three studies reported the use of new imaging modalities for intraoperative diagnostics.

Lopez et al. used confocal laser endomicroscopy to detect tumors as well as damage to the neurovascular bundle. The study revealed the clinical feasibility with standard robotic instrumentation [23]. The frequency of abdominal wall hematoma caused by trocars during insertion for RARP might be decreased through an infrared device that detects veins. In a study of 724 cases, the device led to change in trocar placement in 65% of all cases and decreased the frequency of abdominal wall hematoma from 8.8% to 2.6% (*p* = 0.03) [24]. Bianchi et al. demonstrate the application of augmented reality to perform intraoperative frozen sections. In this study augmented reality was used to guide intraoperative frozen section in 20 patients that were propensity score matched against 20 patients. Positive surgical margins at the level of the index lesion were significantly reduced in the augmented reality guided group (5% vs. 20%, *p* = 0.01) [25].

### 3.2. Intraoperative Detection of Lymph Node Metastases

Intraoperative detection of lymph nodes via specialized imaging has been analyzed in 12 studies identified by our literature search. Thereby three technologies were used, namely fluorescence cameras and drop-in beta and gamma probes. In recent studies PSMA has thereby been used as a target for the fluorescent dye.

Van der Poel et al. revealed the feasibility of an approach to use intraoperative fluorescent imaging to detect SN during RARP. Hereby, the tracer indocyanine-(ICG)-^99m^Tc was injected into the prostate under ultrasound guidance three hours prior to surgery. Two hours after injection, SPECT-CT were acquired to detect SN. Intraoperatively, a fluorescence laparoscope and a laparoscopic gamma probe were used to identify SN. In total, 11 patients underwent this procedure. Fluorescent imaging improved the detection SN in this setting, especially in areas with high background radioactivity [26].

De Korne et al. analyzed whether the site of injection has an impact on detection of SN during surgery. In this study, 67 patients received an ICG-^99m^Tc-nanocolloid injection into the prostate. Intratumoral tracer injection increased the chance of visualizing nodal metastases [29].

KleinJan et al. report a combined approach using indocyanine green-^99m^Tc-nanocolloid as a radioactive and fluorescent tracer. Here, no improvement in detection rates of sentinel lymph nodes was observed. The procedure is described as safe [27]. This tracer was further evaluated by van den Berg et al. They revealed that the combination of ICG-^99m^Tc-nanocolloid together with the lymphangiographic tracer fluorescein improves lymph node detection in patients undergoing RARP [28]. Özkan et al. revealed in a patient cohort of 50 patients that of nine LN positive patients eight had fluorescent positive LN whereas six were detected by preoperative PSMA-PET CT [37].

Another study using indocyanine green-^99m^Tc-nanocolloid revealed higher detection rates of positive lymph nodes in patients undergoing sentinel node biopsy during RARP [32]. A recent phase-II trial analysed the status of indocyanine green-^99m^Tc-nanocolloid further and revealed that intratumoral application improves detection rates compared to application into the prostate. However, metastatic spread from non-index tumors was not detected by the intratumoral application. Therefore, the authors propose combining the intratumoral and intraprostatic tracer injection to optimize sentinel lymph node detection [33]. In a retrospective study, Hinsenveld et al. revealed that the combination of preoperative PSMA PET-CT and ^99m^Tc-nanocolloid for sentinel lymph node detection increased the overall detection in patients with PSMA negative lymph node metastases [30].

Collamati et al. performed a different approach and further develop the approach of SPECT-isotopes by using ^68^Ga-PSMA-11 and a DROP-IN beta particle detector [31]. A comparable approach has been performed by Gondoputro et al. This group used a DROP-IN γ-probe and ^99m^Tc PSMA as a tracer to detect lymph node metastases. This prospective single-arm study (*n* = 12) revealed a high detection rate of positive lymph nodes outside the resection template. A total of 11 metastatic lymph nodes were detected that were not visible on PSMA-PET imaging [34].

Dell’Oglio et al. performed a study to compare a DROP-IN gamma probe with traditional laparoscopic gamma probes as well as fluorescence guidance. Thereby, 47 sentinel lymph node procedures were conducted in the intervention group with 100% detection in the intervention group. Furthermore, 91% of those were identified by fluorescence imaging and 76% by the laparoscopic gamma probe [35].

The sensitivity and specificity of the concept of PSMA guided surgery is currently being tested in a phase II study by Gandaglia et al. In a planned interim analysis, sensitivity (67%), specificity (100%), positive predictive value (100%), and negative predictive values (90%) were observed. Despite an overall good performance, the authors raise the issue of suboptimal sensitivity leading to missed micrometastases of the approach [36].

### 3.3. Training

A total of 15 studies focused on training of surgeons for RARP.

#### 3.3.1. Virtual Training

Various approaches using imaging for virtual- or simulation-based surgical training have been described in 12 studies.

Hung et al. report on one of the first simulators that was still limited to basic skill training [38]. However, Aghazadeh et al. already report a positive correlation between simulated robotic performance and robotic clinical performance [39]. Further virtual reality models are used, and it has been demonstrated that they improve surgical skills of novice surgeons [41].

Shim et al. demonstrate in a study with 45 participants that educational videos are comparable to expert-guided training but are superior to unguided training to fulfil robotic surgical tasks [42]. Shim et al. also investigated the performance of procedure specific training modules in virtual simulators for vesicourethral anastomosis and revealed significant improvements in live surgery after undergoing the training module [43].

Papalois et al. discuss a mixed reality application to train surgical decision making and anatomical structures. Here, multi-rater agreement reached 70.0% for every step of the training and significant improvement was achieved through the training [48].

Basic robotic skills acquired in the lab are transferable to the operating room according to Almarzouq et al. as they observe a positive correlation between the Global Evaluative Assessment of Robotic Skills (GEARS) scores for defined practice sessions on a simulator compared to GEARS scores during urethro-vesical anastomosis and bladder mobilization [44].

Simulation-based training and its effectiveness might depend on the experience level that trainees have. Hoogenes et al. revealed in a randomized trial that two different training programs led to different outcomes in junior trainees but not in more experienced trainees. The hereby used dV-Trainer (dV-T) (Mimic Technologies, Inc., Seattle, WA, USA) uses similar hand and foot controls as a da Vinci console whereas the da Vinci Surgical Skills Simulator (dVSSS) is software that is integrated into the console and uses the normal hand and foot controls [40]. This impact on the learning curve of surgeons is further analyzed by Wang et al. Here, surgeons with VR training revealed shorter learning curves than surgeons without, leading to shorter procedure times and especially anastomosis times (25.1 ± 7.1 min versus 40.0 ± 12.4 min; *p* = 0.015) [45].

A new development is full procedure simulation. Ebbing et al. demonstrate the face and content validity of a full procedure simulation module [47].

Besides from improving surgical skills based on simulation training, Olsen et al. address the question of when to proceed from simulation-based training to live surgery. This research group found a simulator score based on performance during bladder neck dissection, neurovascular bundle dissection and ureterovesical anastomosis that predicts experience levels of surgeons. According to the authors this score might be used to define which surgeon can proceed to supervised clinical training [46].

Further, training scores derived from simulation-based training not only correlate with scores in live surgery but can also impact clinical outcomes as continence recovery rates. In a study from Sanford et al., a high performance during VR needle driving led to a continence recovery rate after 24 months of 98.5% versus 84.9% in surgeons with lower scores (*p* = 0.028) [49].

#### 3.3.2. Peer Review and Structured Feedback

Video review has been identified as an important element of training by van der Leun et al. In this study, students revealed significantly less injuries to the urethra or performed sutures with higher accuracy when reviewing videos of their training [50].

As RARP can be recorded including the use of augmented reality platforms, mentoring and teaching is possible from remote, potentially improving diffusion of robotic training beyond centers [51].

The future in this process might be artificial-intelligence-based video-labeling. A preliminary study of Youssef et al. demonstrates the feasibility of self-training of novices to surgical procedures to perform segmentation of RARP videos [52].

## 4. Discussion

RARP is a challenging procedure that requires precise treatment planning and intraoperative visualization. Various imaging tools have been developed to optimize outcomes of patients with PC. Thereby, the combination of innovative imaging tools and intraoperative guidance on the video console are at the center of the current research. We provide a comprehensive overview of the current literature and insights into future developments.

New imaging technologies can provide assistance at every step of RARP. Treatment of the primary tumor as well as LN dissection can be improved by incorporating those technologies into the surgical workflow as outlined in several feasibility studies described in the results part of this manuscript. Ultimately, surgical training can be enhanced by those advances and might be standardized by the support of simulation-based training and standardized performance metrics in order to improve outcomes [53].

Guidelines have not incorporated most of the described techniques yet. The current EAU guideline describes MRI-guided and PSMA-PET-based normograms that omit the necessity for LN dissection in certain patients. Our review has not covered this topic, as it does not cover staging that is used for both RARP and conventional prostatectomy. Sentinel lymph node biopsy and the use of indocyanin green is discussed in the current guidelines, but insufficient evidence is seen as an obstacle to a broad use of this technology as a meta-analysis revealed a sensitivity of 95.2% and NPV of 98.0% for finding LN metastases in those patients regardless of the primary surgical approach [1,54].

Despite a currently low uptake in guidelines, the impact of current augmented reality models in small case series is dramatic. Around one out of three procedures are performed differently when using the superimposed imaging as described by Schiavina et al. [20]. As the input imaging modalities (MRI, PSMA-PET CT) and robotic systems for RARP become increasingly available [55], the impact of a combination of both technologies might be dramatic for future management of patients with prostate cancer. This impact is not only restricted to the surgical procedure, where preoperative imaging can help to guide surgeons. In addition, preoperative counseling of patients and planning of surgeries can be impacted by virtual reality approaches. However, some adaptions must be made to implement all technologies. To address this, several groups have developed novel tools to provide fluorescence imaging or detection of radioactivity on robotic consoles. However, further efforts will be required to optimize the interfaces between surgeons and the currently developed tools. Interestingly, the addition of a variety of tools to the conventional surgical platforms might change the market leadership of currently dominating companies. Potentially, platforms that are open for development and quickly integrate new cost-effective tools might provide advantages for urologists.

Currently, urologic curricula have no structure for robotic assisted surgery. In the Netherlands, residents already participate in robotic surgery mostly in their final year of residency. However, no criteria exist when residents are allowed to take up training or surgery. At some institutions residents are required to complete online training courses while others are required to reach a certain threshold at simulator-based training [56]. New imaging technologies and combining them with surgical curricula might help to structure the education of future surgeons. As demonstrated in our analysis, those curricula must be designed differently for less experienced and experienced surgeons. New biomarkers can be developed to predict surgical learning curves and to give feedback to surgeons. Interestingly, lifelong training might be possible under those conditions as experienced surgeons can still receive feedback from those algorithms or peer surgeons despite spatial distance.

Financial toxicity has to be considered when adding new armamentarium to the diagnostic and treatment landscape of prostate cancer patients [57]. Adding more features and specialized instruments to a robotic system requires more resources in the healthcare system and might add to the direct costs of RARP that can already be considerable with conventional tools [58]. RARP exceeds the costs of conventional surgery by approximately EUR 2000 [59]. Further equipment such as gamma-probes or preoperative imaging is required for AR and VR augmented approaches. Interestingly, all cited studies in this manuscript do not report on the actual costs of the approach. In addition, not only the direct costs of required technology are of note. When integrating these technologies in a complex metaverse, new infrastructure is required which might not be affordable and thereby might not be provided all over the world [60]. However, some technologies might not necessarily increase the financial toxicity, as especially virtual reality models mostly rely on software and might therefore profit from low material costs and potentially improved outcomes. Future studies will have to assess the impact of new technologies on the overall costs of RARP.

Ultimately, the emerging imaging tools can contribute to a complete change in urologic surgery as it may allow for a step-by-step development towards autonomous surgery. Currently, RARP is performed completely controlled by a surgeon. Current tracking and localization techniques as outlined in the manuscript can help to determine organ boundaries or tumor location. In a first approach this information supports a surgeon to detect those important structures or provides feedback for training. Further development of those techniques might leverage this information and lead to autonomous surgery [61].

The adoption of technology might be impacted by the age of surgeons. Similar to the use of technology in urological patient cohorts [62], age plays a role for the overall use of technology. Technology adoption in physicians is dependent on the age of physicians. However, only very high age is impacting the uptake significantly [63]. Still, there is a need to further investigate the adoption of those new technologies amongst surgeons beyond the feasibility that has been shown in the analyzed studies in this manuscript. In addition, the importance of training and stepwise implementation of the described imaging modalities is paramount [64]

Most of the discussed studies are highly limited by their study design. Mostly, match-pair analysis, retrospective cohort analysis or exploratory single-arm studies are performed. Therefore, a final conclusion regarding the impact of those new technologies on outcomes of patients cannot be drawn and requires further research especially in the light of cost-effectiveness. The authors do not report on direct and indirect costs associated with the introduction of those new technologies. Further, a variety of different instruments and programs are used throughout the studies. Especially for AI-based applications, precise reporting of the algorithm functions will be paramount to facilitate a clear explanation [65]. Standardization and use in several consecutive studies might improve those issues in the future.

## 5. Conclusions

New imaging technologies have been increasingly tested to reduce complications and improve surgical outcomes of patients undergoing RARP. Currently, the feasibility of combined approaches using preoperative imaging or intraoperatively applied radioactive or fluorescent dyes has been demonstrated while a prospective confirmation of improvements is currently ongoing. Balancing improvements in surgical outcomes against financial toxicity of new imaging technologies for RARP will be a cornerstone for a broad clinical implementation.

## Figures and Tables

**Table 1:** Evidence synthesis.

Author [Ref.]	Year	Study Design	LoE [6]	Organ	Area of Application	Imaging Modality
Wake et al. [7]	2019	Prospective	IV	Prostate	Preoperative Planning	MRI—Virtual Reality, 3D models
Shirk et al. [8]	2022	Prospective	II	Prostate	Preoperative Planning	MRI—Virtual reality
Martini et al. [9]	2022	Retrospective	IV	Prostate	Preoperative Planning	MRI—3D models
Checcuci et al. [10]	2022	Retrospective	IV	Prostate	Preoperative Planning	MRI—3D models
Porpilgia et al. [11]	2018	Prospective	IV	Prostate	Visualization of PT	MRI—console
Porpiglia et al. [12]	2018	Prospective	III	Prostate	Visualization of PT	MRI—console
Samei et al. [13]	2018	Prospective	IV	Prostate	Visualization of PT	Ultrasound
Kratiras et al. [14]	2019	Prospective	IV	Prostate	Visualization of PT	MRI—tablet
Porpiglia et al. [15]	2019	Prospective	III	Prostate	Visualization of PT	MRI—console
Porpiglia et al. [16]	2019	Prospective	III	Prostate	Visualization of PT	MRI—console
Mehralivand et al. [17]	2019	Prospective	IV	Prostate	Visualization of PT	MRI—separate display
Canda et al. [18]	2020	Prospective	IV	Prostate	Visualization of PT	MRI/PSMA-PET—console
Samei et al. [19]	2020	Prospective	IV	Prostate	Visualization of PT	MRI/Ultrasound—console
Schiavina et al. [20]	2021	Prospective	IV	Prostate	Visualization of PT	MRI—console
Tanzi et al. [21]	2021	Retrospective	IV	Prostate	Visualization of PT	Console
Padovan et al. [22]	2022	Retrospective	IV	Prostate	Visualization of PT	Console
Lopez et al. [23]	2016	Prospective	IV	Prostate	Intraoperative diagnostics	Confocal
Law et al. [24]	2018	Retrospective	IV	Abdominal wall	Intraoperative diagnostics	Infrared
Bianchi et al. [25]	2021	Prospective	III	Prostate	Intraoperative diagnostics	Augmented reality—console
van der Poel et al. [26]	2011	Prospective	III	LN	Intraoperative detection of LN	Fluorescent camera
KleinJan et al. [27]	2016	Prospective	IV	LN	Intraoperative detection of LN	Fluorescent camera
van den Berg et al. [28]	2017	Prospective	IV	LN	Intraoperative detection of LN	Fluorescent camera
De Korne et al. [29]	2019	Retrospective	III	LN	Intraoperative detection of LN	Fluorescent camera/gamma probe
Hinsenveld et al. [30]	2020	Retrospective	III	LN	Intraoperative detection of LN	Fluorescent camera
Collamati et al. [31]	2020	Prospective	IV	LN	Intraoperative detection of LN	DROP-IN beta particle detector
Mazzone et al. [32]	2021	Retrospective	III	LN	Intraoperative detection of LN	Fluorescent camera
Wit et al. [33]	2022	Prospective	II	LN	Intraoperative detection of LN	Fluorescent camera
Gondoputro et al. [34]	2022	Prospective	IV	LN	Intraoperative detection of LN	Drop-IN gamma detector
DellÓglio et al. [35]	2021	Prospective	IV	LN	Intraoperative detection of LN	Drop-in gamma probe, laparoscopic gamma probe, fluorescent camera
Gandaglia et al. [36]	2022	Prospective	III	LN	Intraoperative detection of LN	Drop-in gamma probe
Özkan et al. [37]	2022	Retrospective	IV	LN	Intraoperative detection of LN	Fluorescent camera
Hung et al. [38]	2011	Prospective	IV	Prostate	Education/ Training	Simulator—basic skills
Aghazadeh et al. [39]	2016	Prospective	IV	Prostate	Education/ Training	Simulator—clinical skills
Hoogenes et al. [40]	2018	Prospective	III	Prostate	Education/ Training	Simulator
Harrison et al. [41]	2018	Prospective	III	Prostate	Education/ Training	Simulator—clinical skills
Shim et al. [42]	2018	Prospective	IV	Prostate	Education/ Training	Video instruction vs. Guided
Shim et al. [43]	2018	Prospective	IV	Prostate	Education/ Training	Simulator
Almarzouq et al. [44]	2020	Prospective	III	Prostate	Education/ Training	Simulator
Wang et al. [45]	2021	Prospective	III	Prostate	Education/ Training	Simulator
Olsen et al. [46]	2021	Prospective	III	Prostate	Education/ Training	Simulator
Ebbing et al. [47]	2021	Prospective	IV	Prostate	Education/ Training	Full procedure simulator
Papalois et al. [48]	2022	Prospective	IV	Prostate	Education/ Training	Mixed reality/VR Glasses
Sanford et al. [49]	2022	Prospective	IV	Prostate	Education/ Training	VR Simulator
Van der Leun et al. [50]	2022	Prospective	III	Prostate	Feedback	Simulator—video
Noël et al. [51]	2022	Prospective	IV	Prostate	Feedback	Remote Teaching
Cheikh et al. [52]	2022	Retrospective	IV	Prostate	Feedback	Video labeling

Abbreviation: LoE: Level of evidence, LN: lymph node, VR: virtual reality.

## Data Availability

Not applicable.
